# *DElite*: a tool for integrated differential expression analysis

**DOI:** 10.3389/fgene.2024.1440994

**Published:** 2024-11-20

**Authors:** Davide Baldazzi, Michele Doni, Beatrice Valenti, Maria Elena Ciuffetti, Stefano Pezzella, Roberta Maestro

**Affiliations:** Unit of Oncogenetics and Functional Oncogenomics (CRO), Centro di Riferimento Oncologico di Aviano (CRO Aviano) IRCCS, Aviano, Italy

**Keywords:** bioinformatics, RNA-seq, differential expression analysis, combination methods, NGS, Software, R package, gene expression

## Abstract

One of the fundamental aspects of genomic research is the identification of differentially expressed (DE) genes between two conditions. In the past decade, numerous DE analysis tools have been developed, employing various normalization methods and statistical modelling approaches. In this article, we introduce *DElite*, an R package that leverages the capabilities of four state-of-the-art DE tools: edgeR, limma, DESeq2, and dearseq. *DElite* returns the outputs of the four tools with a single command line, thus providing a simplified way for non-expert users to perform DE analysis. Furthermore, *DElite* provides a statistically combined output of the four tools, and *in vitro* validations support the improved performance of these combination approaches for the detection of DE genes in small datasets. Finally, *DElite* offers comprehensive and well-documented plots and tables at each stage of the analysis, thus facilitating result interpretation. Although *DElite* has been designed with the intention of being accessible to users without extensive expertise in bioinformatics or statistics, the underlying code is open source and structured in such a way that it can be customized by advanced users to meet their specific requirements. *DElite* is freely available for download from https://gitlab.com/soc-fogg-cro-aviano/DElite.

## 1 Introduction

One of the main goals of transcriptome analysis is to identify significant differences in gene expression patterns between groups or conditions. Differentially expressed (DE) genes are identified based on the extent of variation in gene expression levels between two comparison classes and the statistical significance of this variation. In RNA-sequencing analysis, the expression level of a transcriptomic element is quantified as the number of sequenced fragments aligned to it. Nevertheless, the precise quantification of gene expression and the detection of DE genes are affected by several factors besides sequencing technology, such as gene length and nucleotide composition, sequencing depth, isoforms, overlapping transcripts and cohort size (Zhang et al., 2014). In this regard, the performance of the different tools in pinpointing DE genes in cohorts of small size (e.g. *in vitro* experiments where the comparison classes usually consist of few replicates per condition) is poorly defined. A number of DE analysis tools have been developed based on different mathematical and statistical approaches, either parametric or non-parametric, with the aim of minimizing the impact of these factors. As yet, there is no consensus on the most appropriate approach or algorithm that may yield the most reliable results (Kvam et al., 2012).

Ideally, the integration of different DE tools based on different statistics could help identify the most robust results. However, this can be challenging for users without a strong bioinformatics background. A number of user-friendly suites incorporating different DE tools are available, but these essentially generate descriptive and diagnostic plots rather than performing statistical integration of the results (Nguyen et al., 2024; Liu et al., 2023; Teichman et al., 2023; Sangket et al., 2022; Hunt et al., 2022; Chao et al., 2021; Helmy et al., 2021; López-Fernández et al., 2019; Jiménez-Jacinto et al., 2019; Ge et al., 2018; Li and Andrade, 2017; Varet et al., 2016). The statistical combination of the output of different DE tools by P-value combination methods besides increasing statistical power by combining the summary statistics can allow for the detection of patterns or relationships that may not be apparent through descriptive or diagnostic plots.

Here we present *DElite*, a package developed in the R environment that returns the output of four state-of-the-art DE tools, namely edgeR (Robinson et al., 2010), limma (Ritchie et al., 2015), DESeq2 (Love et al., 2014), and dearseq (Gauthier et al., 2020) with a single command line. To enhance detection capability, *DElite* also provides a combined output of the four tools. Six different statistical methods for combining *p*-values are implemented in *DElite*. Finally, *DElite* produces a report that includes detailed descriptions and explanations of each step, as well as tables and graphs of the different stages of the analysis, thus facilitating the interpretation of the results even for non-expert users. In this work, the different approaches of DE analysis and their integration were cross-examined on datasets of varying sizes. Additionally, *in vitro* validations were carried out to determine their performance in detecting DE genes in small datasets.

## 2 Materials and methods

### 2.1 *DElite* development

The *DElite* package was developed in R v.4.1.2 and also tested with R v.3.6.3 (R Core Team, 2022). *DElite* wraps in a single command line the serial execution of edgeR (Robinson et al., 2010), limma (Ritchie et al., 2015), DESeq2 (Love et al., 2014), and dearseq (Gauthier et al., 2020). Data import and DE analysis follow the developer guidelines of each tool^1^ (Law et al., 2016). The minimum requirements for running *DElite* are the metadata table containing the comparison classes and the quantitative data in the form of a raw count matrix. The standard *DElite* workflow uses default values for filtering thresholds, but these parameters can be customised by the user.

To filter out low-expressed genes and generate filtered counts to be process in parallel by the four tools, *DElite* offers three alternative options: the rowSums function (total sum of the counts attributed to the gene in the entire dataset), the filterByExpr function from edgeR, or a filter based on the gene variance parameter. Filtered counts are then normalized using the normalization method built-in in each tool and DE analysis is conducted. edgeR, limma, and dearseq compute normalization factors via the calcNormFactors function applying the TMM method. Differently, DESeq2 normalize gene counts via the “median of ratios” method. For each normalization strategy, *DElite* provides a series of plots demonstrating the effect of the filtering and normalization phases, and it also calculates the Cook’s Distance (Cook, 1977) for each gene in each sample to identify potential outliers. Moreover, *DElite* generates a number of descriptive plots including MultiDimensional Scaling (MDS), Principal Component Analysis (PCA), volcano plots and heatmaps of DE genes.

Importantly, to improve detection power, *DElite* combines the results from the four tools into a unified output. Specifically, *DElite* re-processes the results of edgeR, limma, DESeq2, and dearseq, by computing the mean of the fold change values returned by each tool. In addition, it calculates a combined *p*-value. The user can select among six different *p*-value combination methods, namely Lancaster’s (Lancaster, 1961), Fisher’s (Mosteller and Fisher, 1948), Stouffer’s (Stouffer et al., 1949), Wilkinson’s (Wilkinson, 1951), Bonferroni-Holm’s (Holm, 1979), Tippett’s (Wishart, 1952). *DElite* also returns the intersection of the genes identified as DE by all four tools, attributing to them the least significant p-value (Max-P). This allows users to identify the most robust observations. The Max-P intersection value and the results from the Lancaster’s combination method are provided by default. Based on recent works that suggest that the Wilcoxon rank-sum test better controls false positives rates when dealing with large datasets (Li et al., 2022), *DElite* provides also the results of this test. Adjusted p-values (padj) are then calculated with the Benjamini–Hochberg correction (Benjamini et al., 2001). Finally, the user can define fold-change and adjusted p-value (padj) thresholds to filter for differentially expressed genes. A comprehensive tutorial is included in the tool and provided as Supplementary File S1.

### 2.2 *DElite* assessment

*DElite* was tested on both synthetic and real-world RNA-sequencing datasets. Synthetic datasets were generated using the generateSyntheticData function from the compcode R package (v.1.30.0) (Soneson, 2014) as described by Soneson et al. (Soneson and Delorenzi, 2013). Three distinct cohorts of different size were generated: a small cohort consisting of three samples per condition (mimicking a common experimental scenario of *in vitro* experiments); a medium-size cohort with ten samples per condition; a large cohort made of 100 samples per condition. For the small and medium cohorts (both covering 12,500 genes), nine distinct types of datasets, approximating a negative binomial distribution, were systematically generated. For each type of dataset, ten independent replicates were produced. Each dataset type featured a different number, magnitude, and direction of genes expected to be scored as DE, and presence or absence of outliers to simulate real world data (Table 1). The large synthetic cohort (100 samples per condition in ten independent replicates) included 20,000 genes, 10% of which were set as DE genes. DE genes were unevenly distributed between the two comparison classes (40% upregulated in one class and 60% in the other). Single and random outliers were introduced using the compcodeR function (Soneson, 2014). Synthetic counts (available upon request) were processed in *DElite*, with features filtered using the filterByExpr function with default parameters. Differential expression was determined using thresholds of padj ≤0.05 and an absolute log2 fold change (|log2(FC)|)≥1 . For each tool and combination method, a confusion matrix was constructed and the performance was assessed using a range of metrics including sensitivity (true positive rate), specificity (true negative rate), F1-score (F1, the harmonic mean of precision and sensitivity), and Matthew’s correlation coefficient (MCC, a measure describing the agreement between predictions and expectations).

**TABLE 1:** This table lists the nine types of synthetic datasets employed to evaluate the performance of DE approaches implemented in *DElite*. NB, Negative Binomial distribution.

#	Generation method	# Of DE genes	Upregulated genes in either class [%]
1	NB Distribution	0	0
2	NB Distribution	1,250	50
3	NB Distribution	1,250	100
4	NB Distribution	4,000	50
5	NB Distribution	4,000	100
6	NB Distribution + Single Outlier	0	0
7	NB Distribution + Single Outlier	1,250	50
8	NB Distribution + Random Outliers	0	0
9	NB Distribution + Random Outliers	1,250	50

*DElite* was also run on real-world data. To this end, we used RNA-sequencing data of cell models of Extraskeletal Myxoid Chondrosarcoma (EMC) (project identifier PRJNA692081 at https://www.ncbi.nlm.nih.gov/sra). EMC is a rare tumor that may express two different fusion transcripts, either EWSR1-NR4A3 (EN) or TAF15-NR4A3 (TN). We recently reported that the expression of EN or TN correlates with a differential activation of axon guidance and semaphorin genes, in both human samples and cell lines (Brenca et al., 2021). On these grounds, we used *DElite* to compare the transcriptome of EN and TN cell lines, four biological replicates each (raw data and results are available in Supplementary File S2). A representative set of semaphorins (SEMA3F, 3G, 4C, 4F, 6D) was selected to validate DE analysis results by RT-qPCR (primers are listed in Supplementary File S3). Features with fewer than 10 total counts (rowsums ≥10) were filtered out. Differential expression was determined with thresholds of padj ≤0.05 and an absolute log2 fold change (|log2(FC)|)≥0.6 . Total RNA extraction, reverse transcription, and RT-qPCR were as previously described (Brenca et al., 2021). The comparative Ct (ΔΔCt) method and the geometric average of two housekeeping genes (GAPDH and β-actin) were used to calculate relative gene expression.

## 3 Results

*DElite* is a novel R package that allows to perform DE analysis based on edgeR, limma, DESeq2, and dearseq tools using a single command line. *DElite* output is designed to be user-friendly and accessible even to users without a strong bioinformatics background. All intermediate and final *DElite* results, including plots and tabular files, are stored into a dedicated directory along with a final report. Besides listing all the steps executed, the report of *DElite* illustrates and describes in detail each step and plot of the analysis.

**FIGURE 1:**
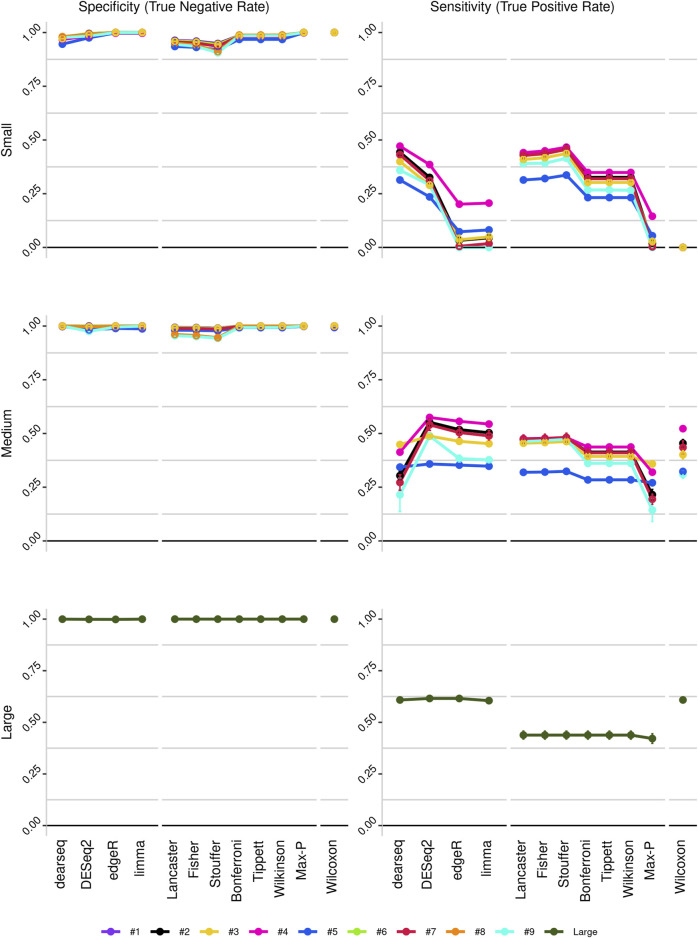
Line plots illustrating the sensitivity and specificity of the different approaches, as derived from the analysis of the three *in silico*-generated datasets: small (cohort size = 3), medium (cohort size = 10) and large (cohort size = 100). Datasets are color-coded, as indicated.

**FIGURE 2:**
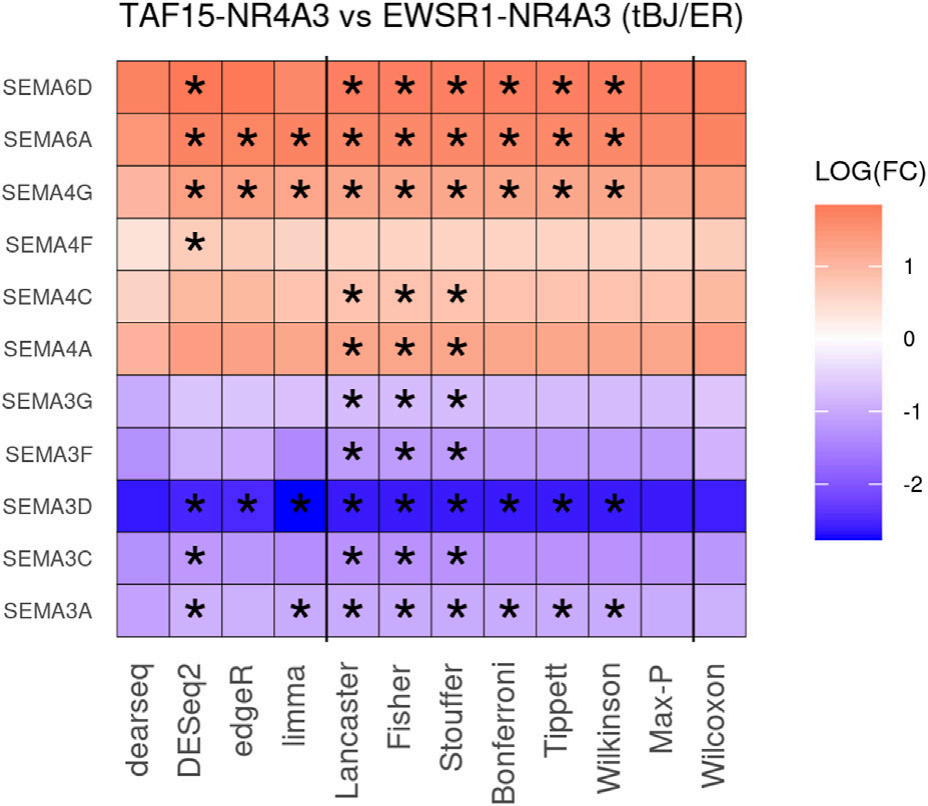
The heatmap depicts the DE of 11 semaphorin genes in TN vs. EN cell models. Asterisks indicate the instances where differential expression was |log2(FC)| ≥ 0.6 and padj ≤0.05.

**FIGURE 3:**
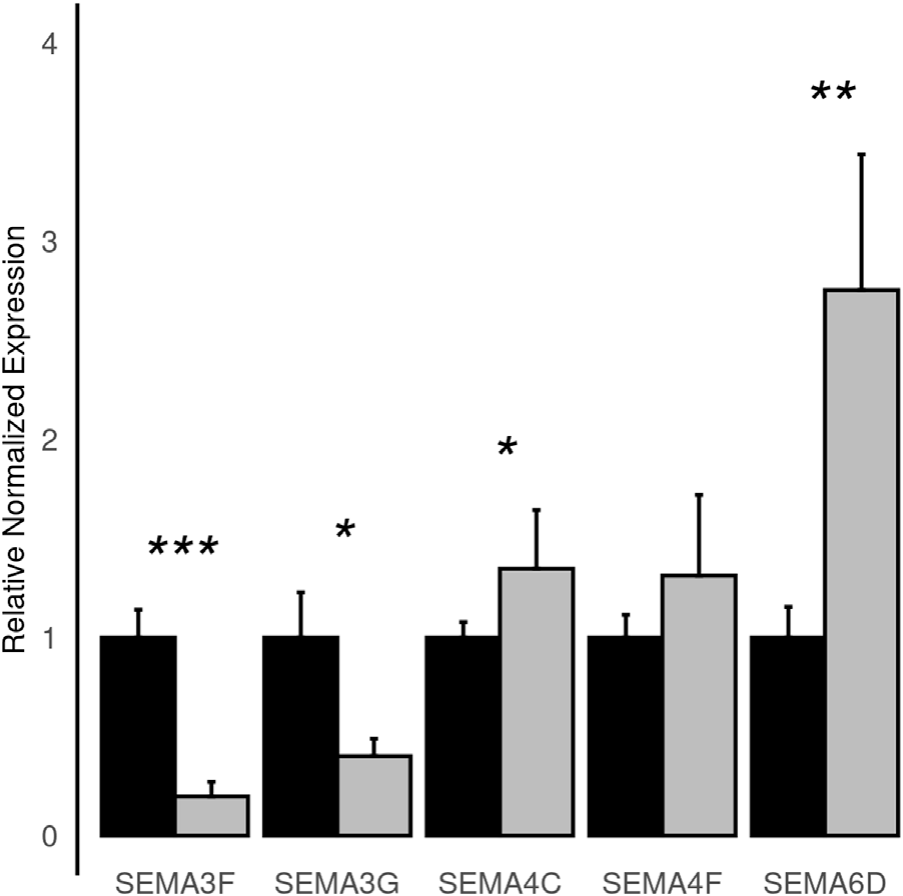
Histograms depicting the normalized relative expression levels of the indicated semaphorin genes as evaluated by RT-qPCR in EN (black) and TN (grey) cells. Statistical significance is as follows: *, p ≤ 0.05; **, p ≤ 0.01; ***, p ≤ 0.00001.

To improve detection capability, *DElite* also returns the intersection (Max-P) and a statistically combined output of the four algorithms. Six different *p*-value statistical combination methods (Lancaster’s, Fisher’s, Stouffer’s, Wilkinson’s, Bonferroni-Holm’s, and Tippett’s) plus the Wilcoxon rank-sum test are implemented in *DElite*. The performance of these combination methods was evaluated on synthetic datasets (Supplementary Table S1). For each tool and combination method, a confusion matrix was constructed and a comprehensive set of metrics was computed to compare the performance of the different approaches (Figure 1; Supplementary Figures S1–S4). Both individual and combined approaches tended to perform better in terms of sensitivity, F1 score, and MCC when datasets were characterized by a relatively even distribution of DE genes between the two classes (for instance, in Figure 1 compare sensitivity for dataset #4, in which of the DE genes, 50% are upregulated and 50% are downregulated in the test class, and sensitivity for dataset #5, in which all the DE genes are upregulated in one class). Individual tools and *DElite* combined results showed comparable specificity, irrespective of cohort size. In medium and large cohorts, individual tools, primarily DESeq2, edgeR and limma, showed overall superior performance in terms of sensitivity, F1 score, and MCC, whereas combination methods performed better than dearseq. We did not observe the claimed improvement (Li et al., 2022) of the Wilcoxon rank-sum test over DESeq2 and edgeR when dealing with large datasets. Noteworthy, combination approaches demonstrated a subtle improvement in sensitivity compared to single tools, especially edgeR and limma, in the analysis of small datasets (Figure 1). This suggests that when dealing with cohorts of limited size, as is often the case in in vitro experiments, a combination approach may be more effective for the identification of DE genes. To address this hypothesis, *DElite* was run on RNA-sequencing data of cell models mimicking the two biological variants, EN and TN, of a rare tumor (EMC) (Brenca et al., 2021). The results of this analysis are reported in Table 2. We focused on semaphorin genes, which have been previously reported to have a major role in the different biology of EN and TN (Brenca et al., 2021). With the exception of dearseq, which failed to call any of the investigated semaphorins as DE, limma, edgeR, DESeq2, and *DElite* combination approaches identified a variable number of DE semaphorins (Figure 2). To provide orthogonal validation of these results, a set of targets, representative of three different outcomes, were evaluated by RT-qPCR. These included SEMA4F, which was detected as DE exclusively by DESeq2; SEMA6D, identified as DE by both DESeq2 and all *DElite* combination methods; SEMA3G, SEMA3F, and SEMA4C, which were exclusively identified as DE by *DElite* combination methods (Lancaster, Fisher, Stouffer). As illustrated in Figure 3, the differential expression of SEMA4F, detected exclusively by DESeq2, was not confirmed by RT-qPCR. In contrast, all the SEMAs identified by *DElite* combination methods, including the one detected also by DESeq2 (SEMA6D), were confirmed by RT-qPCR to be expressed in a statistically different manner by the two cell models. Taken together, these results support the notion that the integration of diverse DE algorithms, through the use of *p*-value combination methods, may increase the sensitivity in detecting DE genes, particularly when dealing with cohorts of small size.

**TABLE 2:** DE analysis in TN vs. EN cell models (padj ≤0.05, |log (FC)| ≥ 0.6). The number of upregulated (UP) and downregulated (DOWN) genes are indicated.

		UP	DOWN
Individual tool	dearseq	0	0
	edgeR	460	426
	DESeq2	813	1,571
	Limma	501	388
Combination method	Lancaster	2,620	4,398
	Fisher	2,772	5,027
	Stouffer	3,080	6,617
	Tippett	589	698
	Bonferroni	588	696
	Wilkinson	589	696
	Max-P	0	0

## 4 Discussion

This study presents *DElite*, a R package for DE analysis that offers user-friendly functionalities, accompanied by a detailed report. The main advantage of *DElite* is that it enables the execution of DE analysis with four state-of-the-art tools (edgeR, limma, DEseq2, and dearseq) with just a single command line. Moreover, *DElite* presents the additional functionality of combining the results of the four algorithms in a statistically controlled manner, unlike other packages that offer the possibility of running different DE analysis tools, but in most cases only generate descriptive and diagnostic plots (Supplementary Table S2) (Nguyen et al., 2024; Liu et al., 2023; Teichman et al., 2023; Sangket et al., 2022; Hunt et al., 2022; Chao et al., 2021; Helmy et al., 2021; López-Fernández et al., 2019; Jiménez-Jacinto et al., 2019; Ge et al., 2018; Li and Andrade, 2017; Varet et al., 2016). To our knowledge, only ExpressAnalystR and RCPA provide a statistically combined output of the implemented tools. ExpressAnalystR relies on two *p*-value combination methods, whilst RCPA on six. Nevertheless, unlike *DElite*, which can be launched with a single command line, these tools require multiple command inputs, do not provide a final analysis report, and use only parametric approaches (DESeq2, edgeR, limma). As observed on both synthetic and real-world data, *DElite* statistical combination methods appear to improve sensitivity over individual tools, particularly when dealing with small datasets.

The current version of *DElite* is based on the four DE algorithms that represent the today’s state-of-the-art for bulk RNA-sequencing data analysis. However, we are committed to further improving it by integrating additional tools as well as pipelines for single-cell RNA-sequencing data analysis.

## Data Availability

The data used in the study were deposited in the SRA repository, accession number PRJNA692081 (https://www.ncbi.nlm.nih.gov/sra/?term=PRJNA692081).

## Footnotes

1
https://bioconductor.org/packages/release/bioc/vignettes/edgeR/inst/doc/edgeRUsersGuide.pdf; http://bioconductor.org/packages/devel/bioc/vignettes/DESeq2/inst/doc/DESeq2.html; https://www.bioconductor.org/packages/devel/bioc/vignettes/dearseq/inst/doc/dearseqUserguide.html

## Supplementary material

SUPPLEMENTARY FIGURE S1 Confusion matrices reporting true positives, false positives, false negatives, and true negatives computed from the results of the different DE tools and combination methods implemented in DElite applied to small **(A)** and medium **(B)** size datasets. A gradient color scale, from best (green) to worst (red), is used. Numbers within each cell represent the sum of the output of ten independent replicates.

SUPPLEMENTARY FIGURE S2 Line plots illustrating the false negative rate and false positive rate of the different approaches, as derived from the analysis of the three in silico-generated datasets: small (cohort size = 3), medium (cohort size = 10) and large (cohort size = 100). Datasets are color-coded, as indicated.

SUPPLEMENTARY FIGURE S3 Line plots illustrating the F1-score and Matthews’s Correlation Coefficient of the different approaches, as derived from the analysis of the three in silico-generated datasets: small (cohort size = 3), medium (cohort size = 10) and large (cohort size = 100). Datasets are color-coded, as indicated.

SUPPLEMENTARY FIGURE S4 Line plots illustrating the accuracy and balanced accuracy of the different approaches, as derived from the analysis of the three in silico-generated datasets: small (cohort size = 3), medium (cohort size = 10) and large (cohort size = 100). Datasets are color-coded, as indicated.
