# Supplementary figures and images for: DElite: a tool for integrated differential expression analysis
Source: Front Genet. 2024 Nov 20;15:1440994. doi: 10.3389/fgene.2024.1440994 (PMC11614847; doi:10.3389/fgene.2024.1440994)

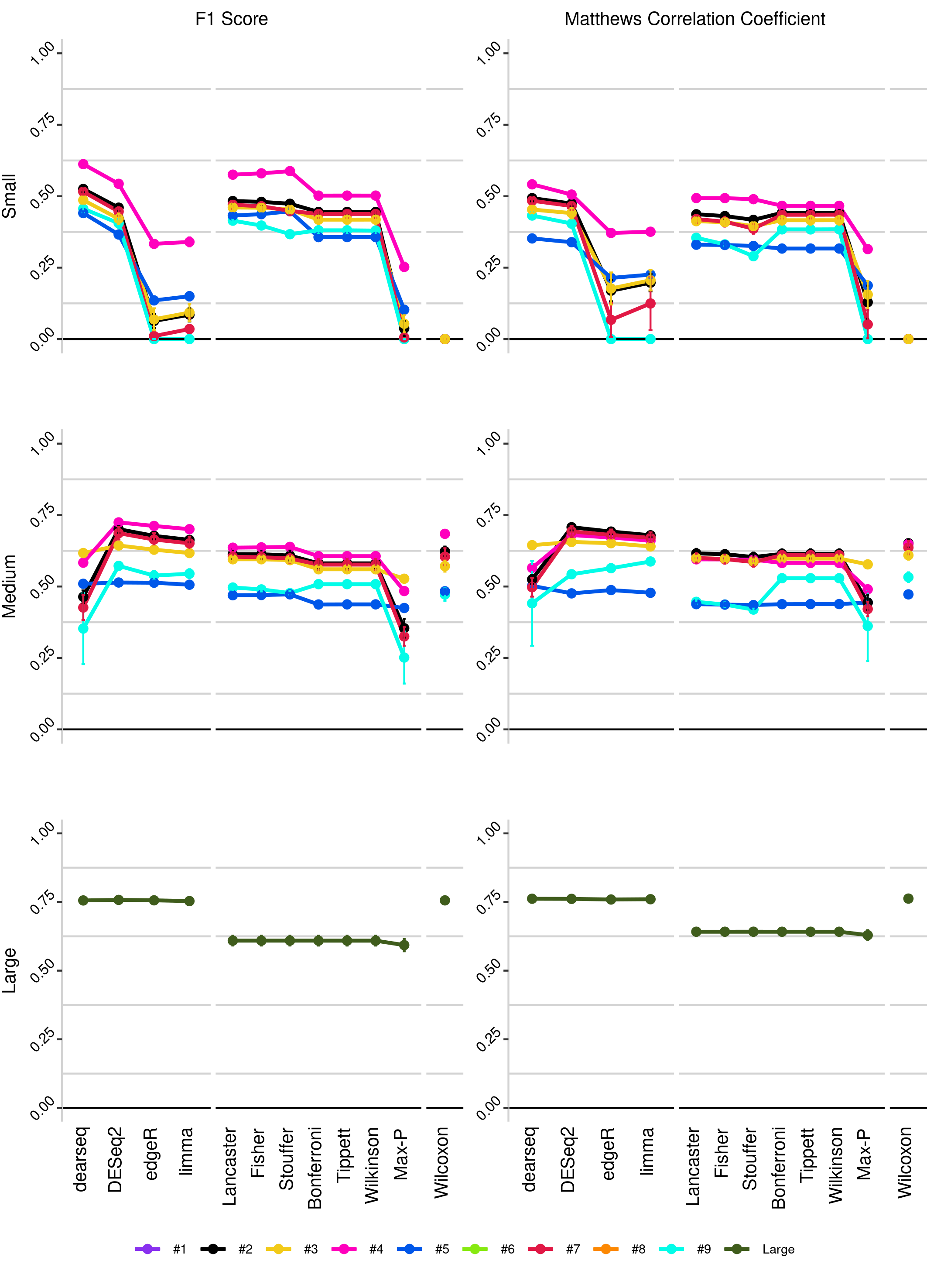

Supplement: Supplementary file 1 [file Image3.tiff]

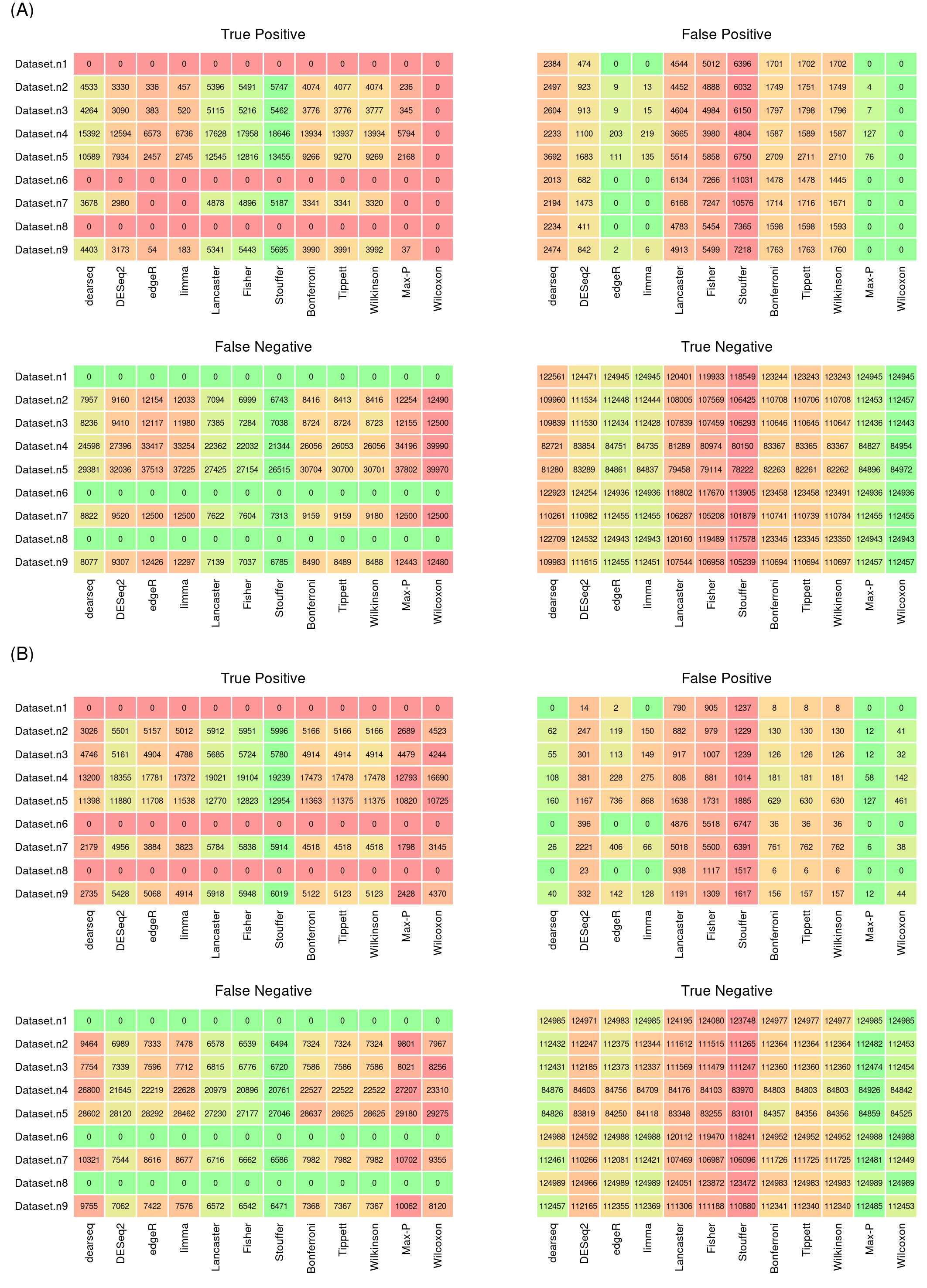

Supplement: Supplementary file 3 [file Image1.tiff]

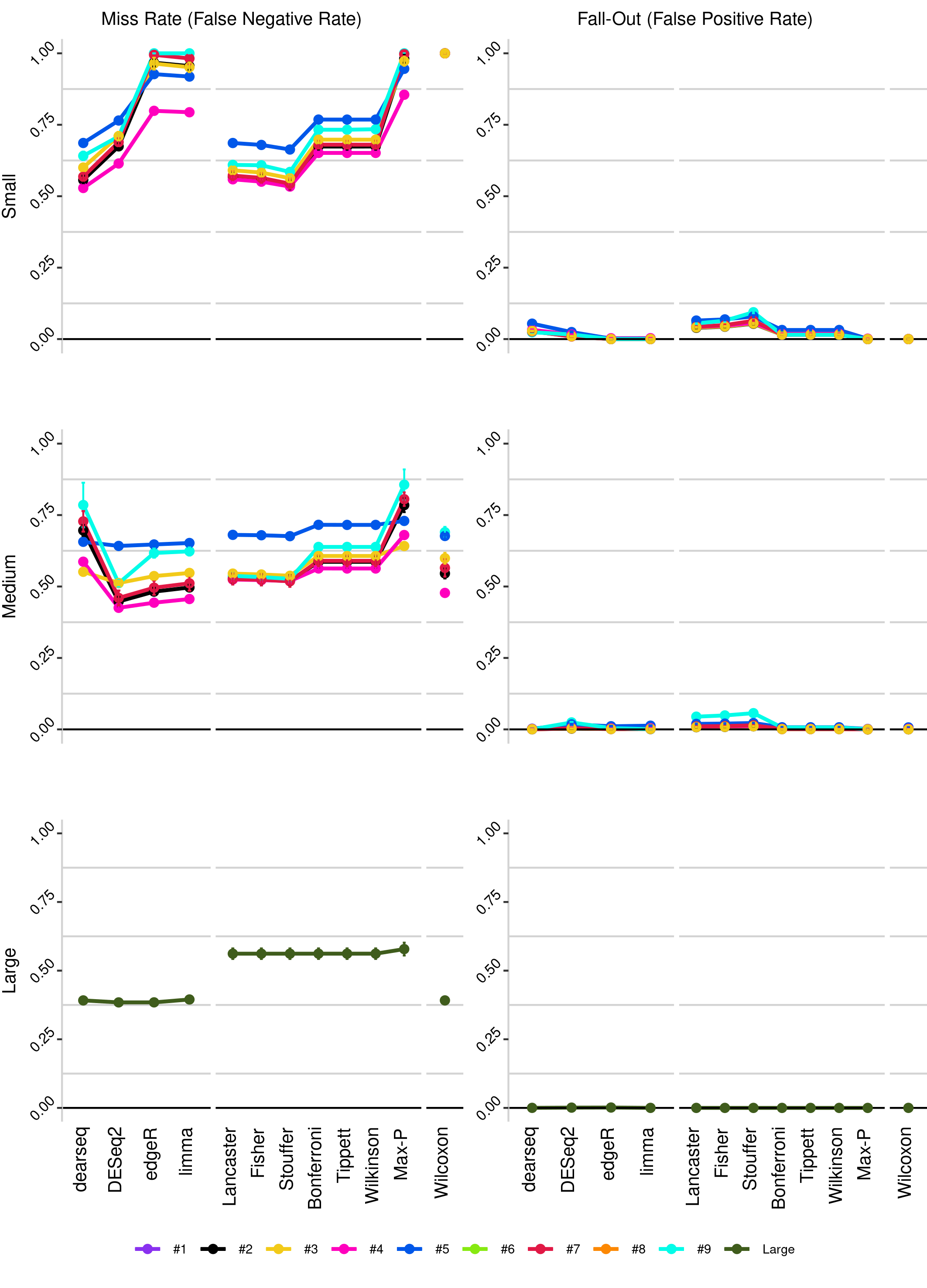

Supplement: Supplementary file 8 [file Image2.tiff]

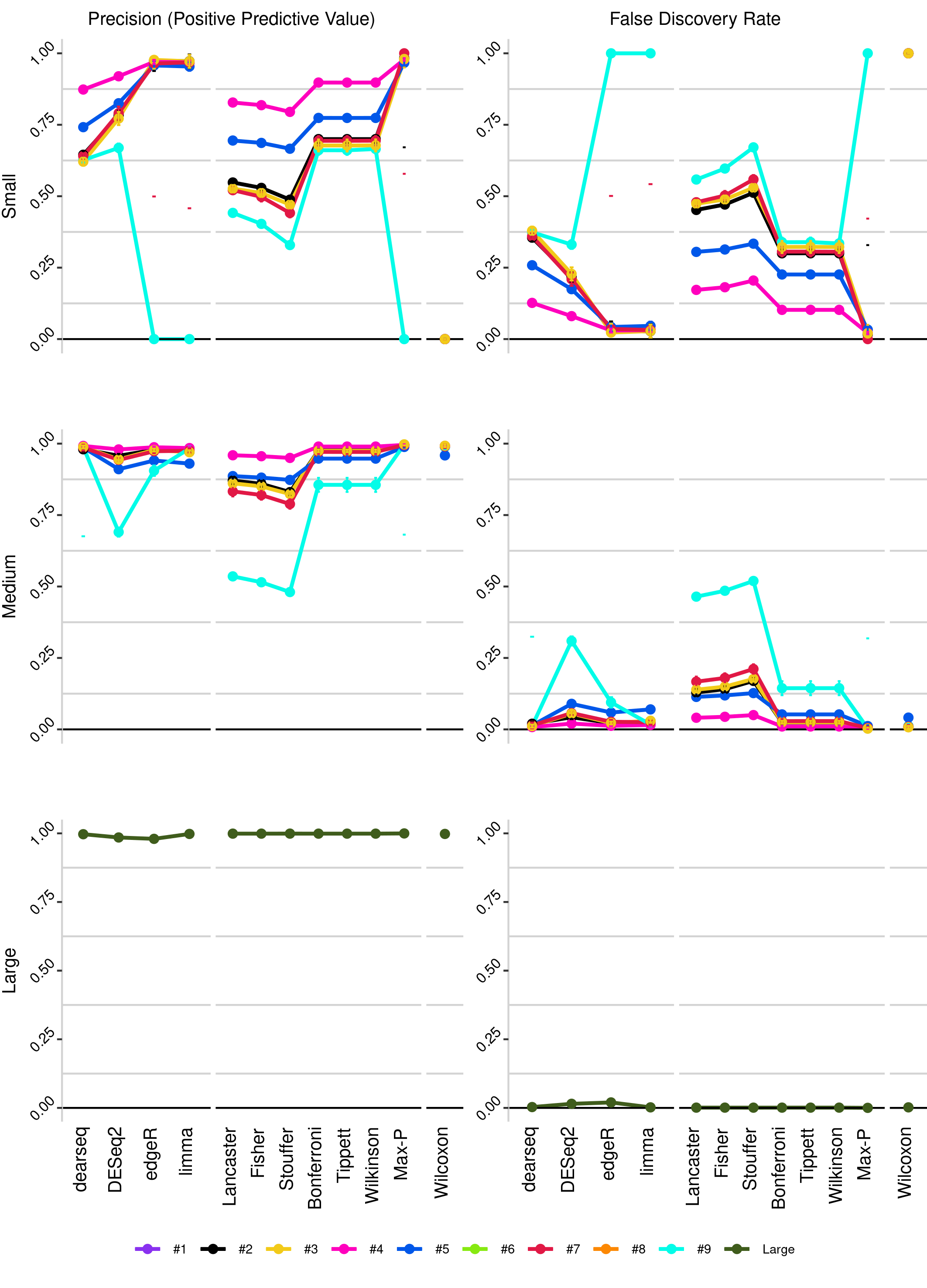

Supplement: Supplementary file 9 [file Image4.tiff]
