# Supplementary material for: DElite: a tool for integrated differential expression analysis
Source: Front Genet. 2024 Nov 20;15:1440994. doi: 10.3389/fgene.2024.1440994 (PMC11614847; doi:10.3389/fgene.2024.1440994)
Supplement: Supplementary file 4 [file DataSheet3.docx]

Supplementary File 3

**RT-qPCR primers.**

GAPDH-FW: 5’ TCACCAGGGCTGCTTTTAAC 3’

GAPDH-REV: 5’ TGACAAGCTTCCCGTTCTCA 3’

β-actin-FW: 5’ CACCTTCTACAATGAGCTGCGT 3’

β-actin-REV: 5’ AGCCTGGATAGCAACGTACATG 3’

SEMA3F-FW: 5’ CGCGAGCCCCTCATTATACA 3’

SEMA3F-REV: 5’ TGACGAAGTTCCCACACTCG 3’

SEMA3G-FW: 5’ GAGGCAGAGGATGGGACCTA 3’

SEMA3G-REV: 5’ GCCTGGAGAGCGATGACTTT 3’

SEMA4C-FW: 5’ GTTTGACCAGGAGCCCATGA 3’

SEMA4C-REV: 5’ GGACACAGTCTGCACAGGAG 3’

SEMA4F-FW: 5’ GCAGGATGTTGCTGTGCTTCGA 3’

SEMA4F-REV: 5’ CTGTCCGAATGTCTTGTGGTCG 3’

SEMA6D-FW: 5’ GCATCTCGTGACCCGTATTGTG 3’

SEMA6D-REV: 5’ CCTAGATGAGCTGTGTTGCCGA 3’
