# Supplementary material for: DElite: a tool for integrated differential expression analysis
Source: Front Genet. 2024 Nov 20;15:1440994. doi: 10.3389/fgene.2024.1440994 (PMC11614847; doi:10.3389/fgene.2024.1440994)
Supplement: Supplementary file 5 [file DataSheet1.pdf]

User guide to **DElite** R package

Dr. Baldazzi Davide, Dr. Doni Michele, Dr. Pezzella Stefano, Dr. Valenti Beatrice, Dr. Ciuffetti Maria Elena, Dr. Maestro Roberta

2024-08-24

Abstract

DElite returns the outputs of the four tools with a single command line, thus providing a simplified way for non-expert users to perform DE analysis. Furthermore, DElite provides a statistically combined output of the four tools, and in vitro validations support the improved performance of these combination approaches for the detection of DE genes in small datasets. Finally, DElite offers comprehensive and well-documented plots and tables at each stage of the analysis, thus facilitating result interpretation. Although DElite has been designed with the intention of being accessible to users without extensive expertise in bioinformatics or statistics, the underlying code is open source and structured in such a way that it can be customized by advanced users to meet their specific requirements. DElite package version: 1.1

INTRODUCTION

DElite is an R package that leverages the capabilities of four commonly used tools for Differential Expression (DE) Analysis.

1. DESEq2 (Love et al. 2014)
2. edgeR (Robinson et al. 2010)
3. Limma-Voom (Ritchie et al. 2015; Law et al. 2014)
4. dearseq (Gauthier et al. 2020)

DElite returns the outputs of the four tools with a single command line, providing a simplified way for non-expert users to cross-examine different approaches of DE analysis. To help pinpointing the most reliable observations, **DElite** also provides the intersection (Max-P method) of the results from the four tools and a statistically combined output using one of the following methods according to user choice.

1. Bonferroni-Holm's Method (Holm 1979)
2. Fisher's Method (Fisher 1925)
3. Lancaster's Method (Inverted Chi-Square) (Lancaster 1961)
4. Stouffer's Method (Stouffer et al. 1949)
5. Tippett's Method (Tippett 1931)
6. Wilkinson's Method (Wilkinson 1951)

USAGE

The **DElite** package provides a simplified way for non-expert user to perform DE analysis. Users can load the DElite package as follow:

```
library(DElite)
```

Users can run a complete analysis with **DElite** by specifying just three required arguments: counts data, metadata, and the condition to test for differentially expressed genes:

```
DElite(counts_file, metadata_file, condition)
```

The **REQUIRED arguments** that the user must specify are:

counts_file Path to un-normalized counts file. Rows refer to genes while columns refer to samples. Header row is required and first column must include features names.

metadata_file Path to metadata file. One of the column name must match with the condition to test for DE genes. First column must include sample names.

condition Condition to test for DE genes. Column name in the metadata file to test for DE genes. Reference level is determined by alphabetical order.

The **OPTIONAL arguments** that the user can specify are:

path Path to the directory where the DElite output folder will be generated. DElite will create a directory with the following naming schema: `DElite_yy-mm-dd_rndstring`

Default is R current working directory

pvalue Significance Threshold for the p-value corrected for multiple testing. Applied in the results filtering phase.

Default value is 0.05

logfc Significance Threshold for the absolute value of the log2 fold change. Applied in the results filtering phase.

Default value is 1

lowcounts Low counts filtering method. One of "rowsums", "var" and "fbexp". The "rowsums" method filters genes based on the sum of their counts across all samples. The "var" method filters genes based on the variance of their counts distribution. The "fbexp" method utilizes the `filterByExpr` function from the edgeR package.

Default is "fbexp"

fbexp Threshold value to filter out low counts via `filterByExpr` function. Applied only when `lowcounts="fbexp"`

Default value is 10

rowsums Threshold value to filter out low counts via rowsums function. Applied only when `lowcounts="rowsums"`

Default value is 10

var Threshold value to filter out low counts via variance filter. User submitted value has to be interpreted as the top quantile percentage retained. Applied only when `lowcounts="var"`

Default value is 0.25

wilcoxon Boolean, either T or F (TRUE or FALSE). Run the Wilcoxon rank-sum test if TRUE.

Default is F

combine P-value combination method applied by DElite during the meta-analysis. Options are: "bonferroni", "fisher", "lancaster", "stouffer", "tippett", "wilkinson". DElite will always perform in addition to the method chosen the "max" method.

Default is "lancaster"

dearseq_mode dearseq analysis approach, choose between "permutation" or "asymptotic". "permutation" mode is suggested when number of samples is less than 10.

Default "asymptotic"

To test and explore DElite, please run the following commands. A sample dataset is already included for your convenience.

```
counts_file = system.file("extdata", "counts_pruned.csv", package="DElite", mustWork=TRUE)
metadata_file = system.file("extdata", "metadata.csv", package="DElite", mustWork=TRUE)
condition = "condition"
DElite::DElite(counts_file, metadata_file, condition)
```

TUTORIAL

Here we provide an example analysis. For your convenience a sample data is already included.

1) Specify the profile expression matrix

The counts_file indicates the path (that is a string of characters used to uniquely identify a location in a directory structure) of the raw counts file to be analyzed with DElite.

```
counts_file <- "/path/to/your/counts_file.csv"
```

(!) Tips & Tricks If you don't know how to obtain the path of your file, right click on it, press properties and copy the string right after "Path" or "Parent folder" by selecting it and pressing the keyboard keys "Ctrl" and "C". On the other hand, if you are using RStudio you can navigate through the directories in the "Files" panel. Once you reached the folder where your chosen file is, click the Gear icon "More", and select "Copy folder path to clipboard".

Here we provide an example using an expression profile matrix already included in the package.

```
counts_file <- system.file("extdata", "counts_pruned.csv", package="DElite", mustWork=TRUE)
head(read.csv(counts_file, header=TRUE, sep=",", check.names=FALSE, row.names=1))
```

```
##    sample1 sample2 sample3 sample4 sample11 sample12 sample13 sample14
## g1     593    1001    1215     445     4876     4221     3080    25383
## g2      73     486      34      39      135       27      122      195
## g3      11      36      14      36      104       98       12       53
## g4       2       8      28       3       11        4        0        7
## g5     141     244     254      71      441      768     1541     1114
## g6      16      22      12      14       66      100       94       38
```

As you can see, your expression profile matrix must include feature names in the first column and sample names in the first row.

2) Specify the metadata

Specify the metadata as you previously did for the expression profile matrix.

```
metadata_file <- "/path/to/your/metadata_file.csv"
```

Here we provide an example of metadata included in the package.

```
metadata_file <- system.file("extdata", "metadata.csv", package="DElite", mustWork=TRUE)
head(read.csv(metadata_file, header=TRUE, sep=" ", check.names=FALSE, row.names=1))
```

```
##           condition depth.factor
## sample1           A    0.7729433
## sample2           A    0.8217464
## sample3           A    0.8019498
## sample4           A    0.7258261
## sample11          B    0.8981939
## sample12          B    1.1341414
```

As you can see, metadata must have an header and must report sample names in their first column.

3) Run DElite

Once set up your `counts_file` and the `metadata_file` the only thing left to do is to select the condition to test for differential expression. It must be the name of one of the columns in the metadata file.

```
condition <- "your_testing_condition"
```

In the metadata included in **DElite**, the testing column condition is called “condition”.

```
condition <- "condition"
```

To test and run **DElite** analysis simply type the following:

```
DElite::DElite(counts_file, metadata_file, condition)
```

In order to personalize the analysis, the user can change the optional arguments previously described.

The most basic script in R to run **DElite** should look like this:

```
counts_file <- "/path/to/your/counts_file.csv"
metadata_file <- "/path/to/your/metadata_file.csv"
condition <- "your_testing_condition"
DElite::DElite(counts_file, metadata_file, condition)
```

The **DElite** output directory structure should look like this:

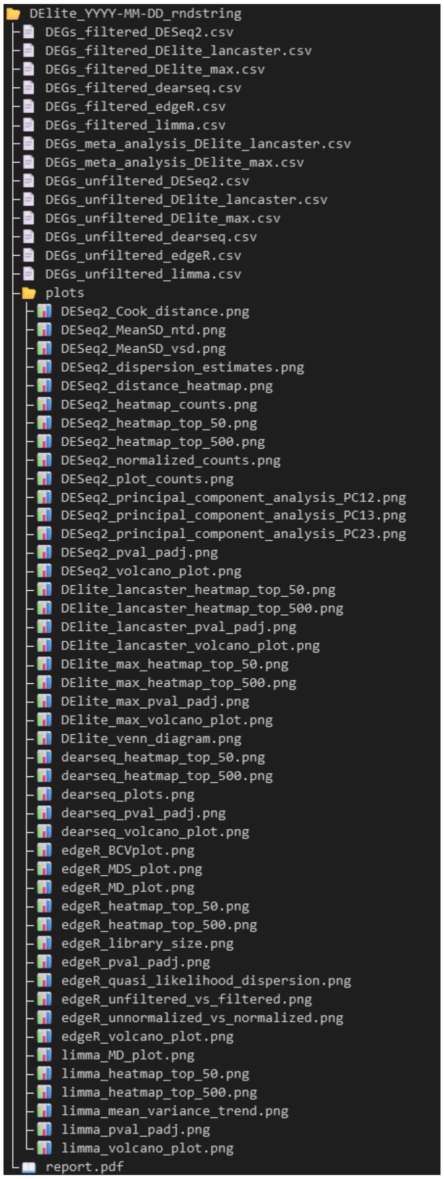

Figure 1: DElite directory structure. Inside the main folder there is the final report in pdf format and all the tables containing the results of the different tools run, both in their unfiltered and filtered version (according user significance thresholds). Inside the plots directory there are all the plots produced by DElite that are included in the report file. All files are named according to the tool or method that are referring to.

Here some of the plots generated by the DElite analysis:

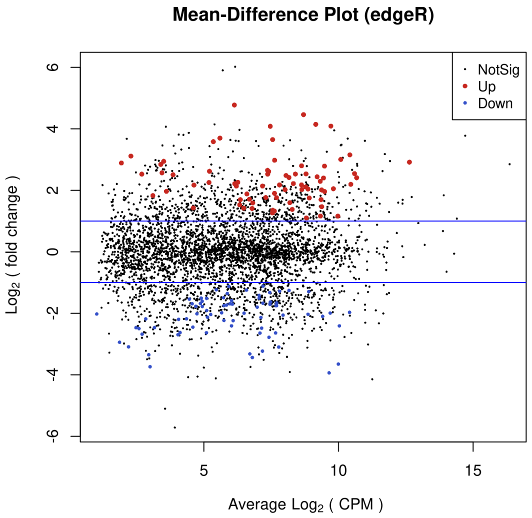

Figure 2: MD plot. Red and blue dots indicate the genes emerged as differentially expressed in the dataset. Red dots resulted as up-regulated while blue dots emerged as down-regulated. Horizontal lines mark the chosen threshold for the |log 2 (FC)|

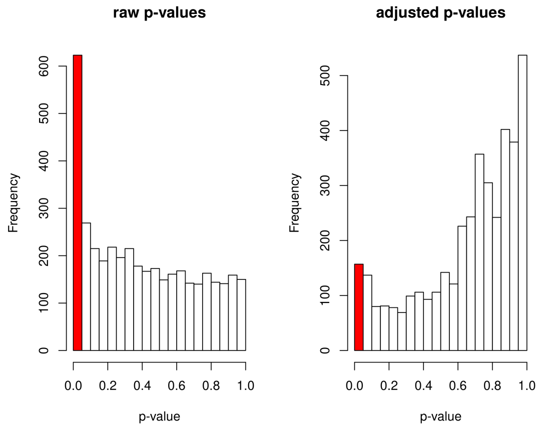

Figure 3: Histograms representing the distribution of p-values (left plot) and adjusted p-values (right plot) respectively. Probability values below the user selected threshold are highlighted in red.

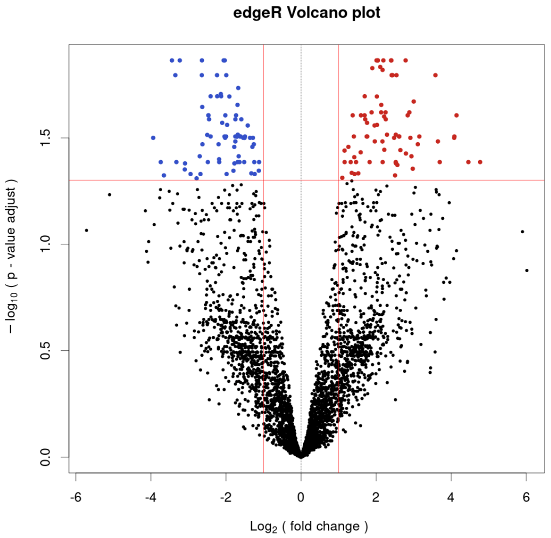

Figure 4: This volcano plot shows genes as the relation between their significance log10(p-value adjust) and log2(FC). Red and blue data points represent genes that passed the filtering criteria. Red genes emerged up-regulated given the tested condition while blue genes emerged as down-regulated.

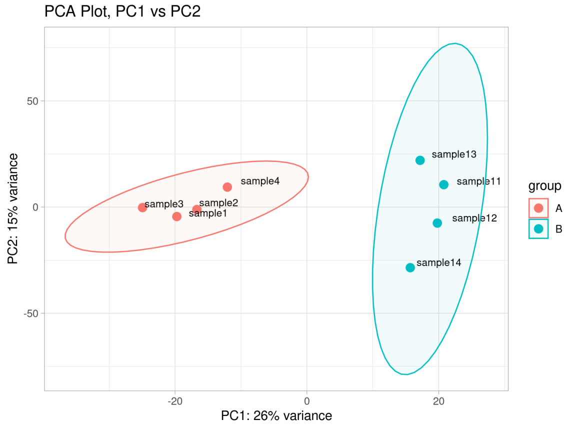

Figure 5: Principal Component Analysis plot representing the Principal components 1 and 2. Samples are labeled according to the condition they belong to.

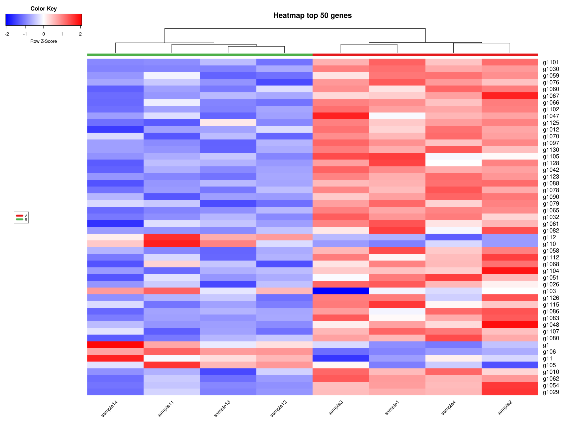

Figure 6: Heatmap representing expression levels of the top 50 DE features.

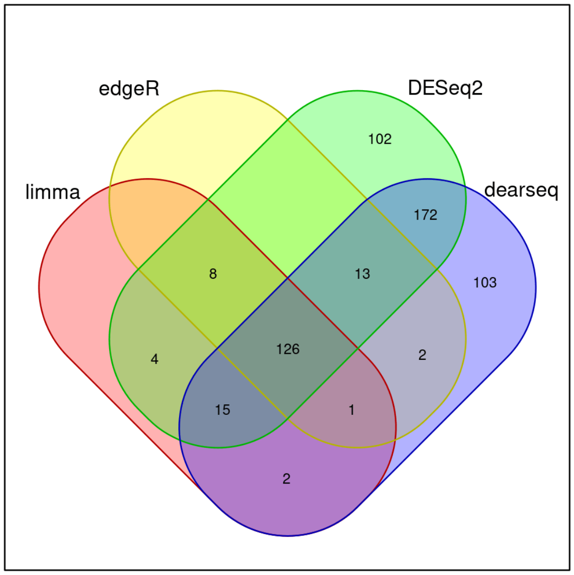

Figure 7: Venn diagram representing the intersection of the results produced by the tools DESeq2, edgeR, limma and dearseq.

Session Info

```
## R version 4.1.2 (2021-11-01)
## Platform: x86_64-pc-linux-gnu (64-bit)
## Running under: Ubuntu 22.04.3 LTS
##
## Matrix products: default
## BLAS:   /usr/lib/x86_64-linux-gnu/openblas-pthread/libblas.so.3
## LAPACK: /usr/lib/x86_64-linux-gnu/openblas-pthread/libopenblasp-r0.3.20.so
##
## locale:
##  [1] LC_CTYPE=en_US.UTF-8       LC_NUMERIC=C
##  [3] LC_TIME=en_US.UTF-8        LC_COLLATE=en_US.UTF-8
##  [5] LC_MONETARY=en_US.UTF-8    LC_MESSAGES=en_US.UTF-8
##  [7] LC_PAPER=en_US.UTF-8       LC_NAME=C
##  [9] LC_ADDRESS=C               LC_TELEPHONE=C
## [11] LC_MEASUREMENT=en_US.UTF-8 LC_IDENTIFICATION=C
##
## attached base packages:
## [1] stats     graphics  grDevices utils     datasets  methods   base
##
## other attached packages:
## [1] DElite_1.1    ggplot2_3.5.1 hexbin_1.28.2 dearseq_1.6.0
##
## loaded via a namespace (and not attached):
##  [1] pillar_1.9.0        compiler_4.1.2      tools_4.1.2
##  [4] statmod_1.5.0       digest_0.6.35       lattice_0.20-45
##  [7] evaluate_0.23       lifecycle_1.0.4     tibble_3.2.1
## [10] gtable_0.3.5        viridisLite_0.4.2   pkgconfig_2.0.3
## [13] rlang_1.1.3         Matrix_1.5-3        DBI_1.1.3
## [16] cli_3.6.2           rstudioapi_0.14     patchwork_1.2.0.9000
## [19] yaml_2.3.8          parallel_4.1.2      xfun_0.43
## [22] fastmap_1.1.1       withr_3.0.0         dplyr_1.1.4
## [25] knitr_1.46          mitools_2.4         generics_0.1.3
## [28] vctrs_0.6.5         grid_4.1.2          tidyselect_1.2.1
## [31] glue_1.7.0          R6_2.5.1            fansi_1.0.6
## [34] pbapply_1.7-0       survival_3.4-0      rmarkdown_2.26
## [37] magrittr_2.0.3      splines_4.1.2       scales_1.3.0
## [40] htmltools_0.5.8.1   matrixStats_0.63.0  colorspace_2.1-0
## [43] utf8_1.2.4          KernSmooth_2.23-20  survey_4.1-1
## [46] munsell_0.5.1
```
